# Supplementary material for: Fluorescent Molecularly Imprinted Polymers Loaded with Avenanthramides for Inhibition of Advanced Glycation End Products
Source: Polymers (Basel). 2023 Jan 20;15(3):538. doi: 10.3390/polym15030538 (PMC9920636; doi:10.3390/polym15030538)
Supplement: Supplementary file 1 [file polymers-15-00538-s001.zip › polymers-2091045-supplementary.pdf]

Supporting Information

Fluorescent molecularly imprinted polymers loaded with avenanthramides for inhibition of advanced glycation end products

Pei Zhu, Huilin Liu*, and Baoguo Sun

Beijing Technology and Business University, 11 Fucheng Road, Beijing, 100048, China.

*Corresponding author: Huilin Liu

Tel: (86 10) 68984545;

Fax: (86 10) 68985456;

Email: liuhuilin@btbu.edu.cn

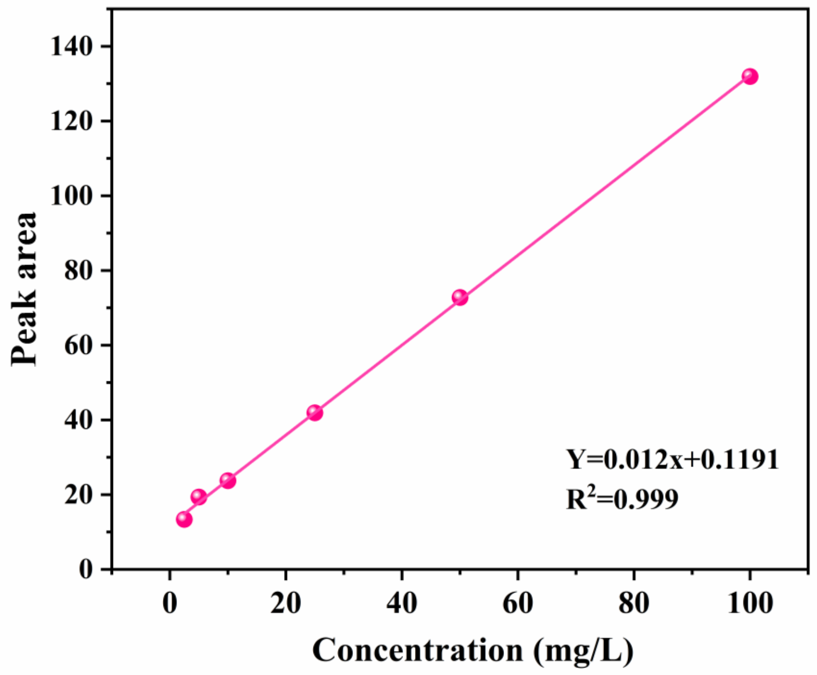

Figure S1. Standard curves of different concentrations of AVAs 2f.

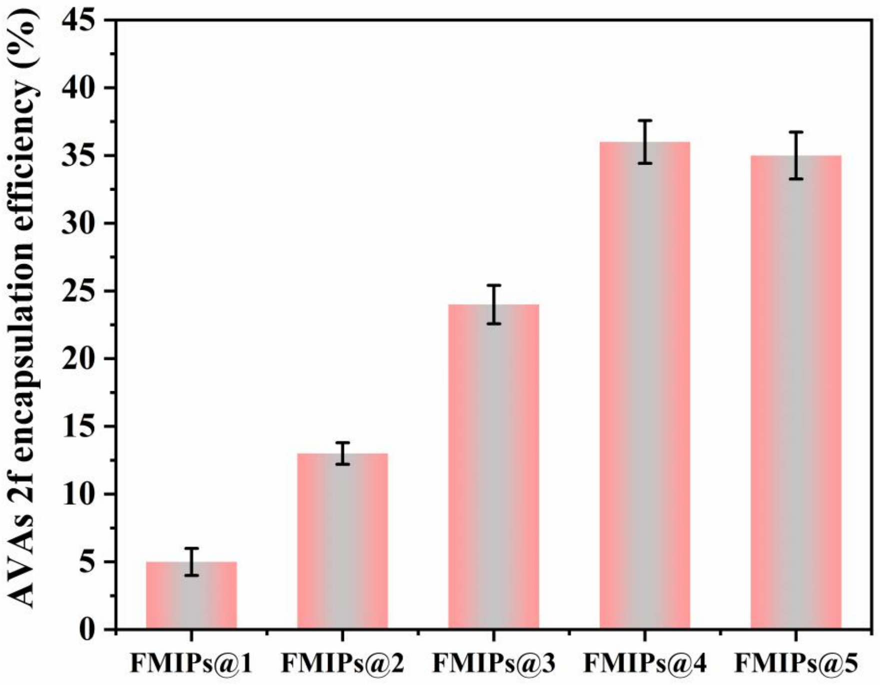

Figure S2. The loading content of AVAs 2f for different FMIPs samples.

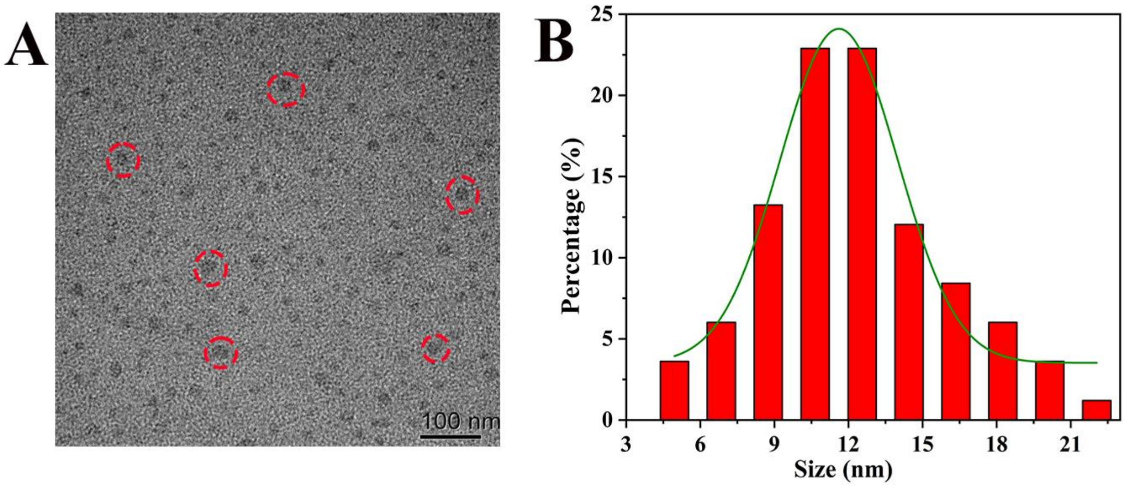

Figure S3. (A) TEM image of CDs. (B) Size distribution of CDs.

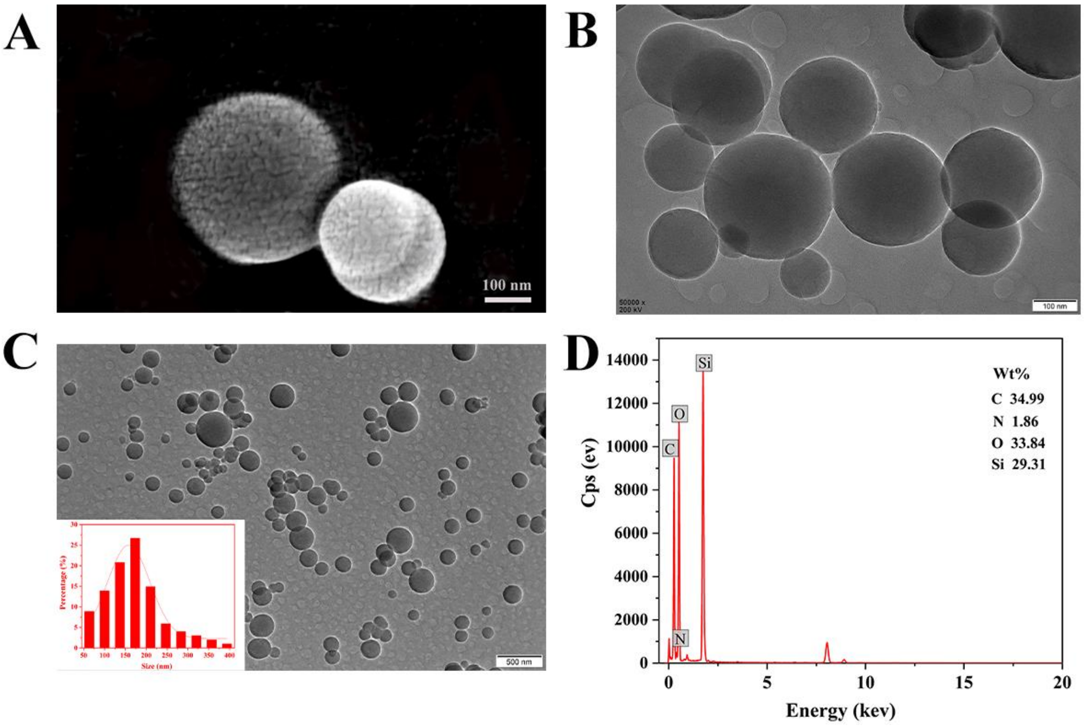

Figure S4. (A) SEM image of FMIPs without AVAs 2f. (B) TEM image of FMIPs without AVAs 2f (with the scale of 100 nm). (C) TEM image of FMIPs without AVAs 2f (with the scale of 500 nm; inset: size distribution of FMIPs without AVAs 2f). (D) EDS spectrum of FMIPs without AVAs 2f.

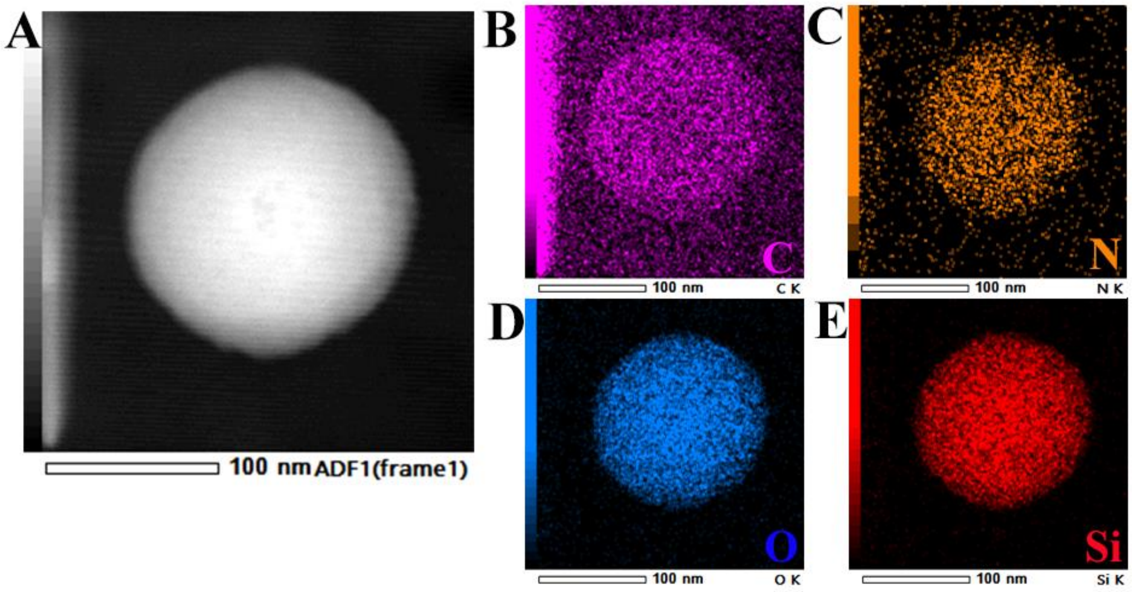

Figure S5. (A) EDS images of FMIPs without AVAs 2f. (B) EDS images of C, (C) N, (D) O and (E) Si elements in FMIPs without AVAs 2f.

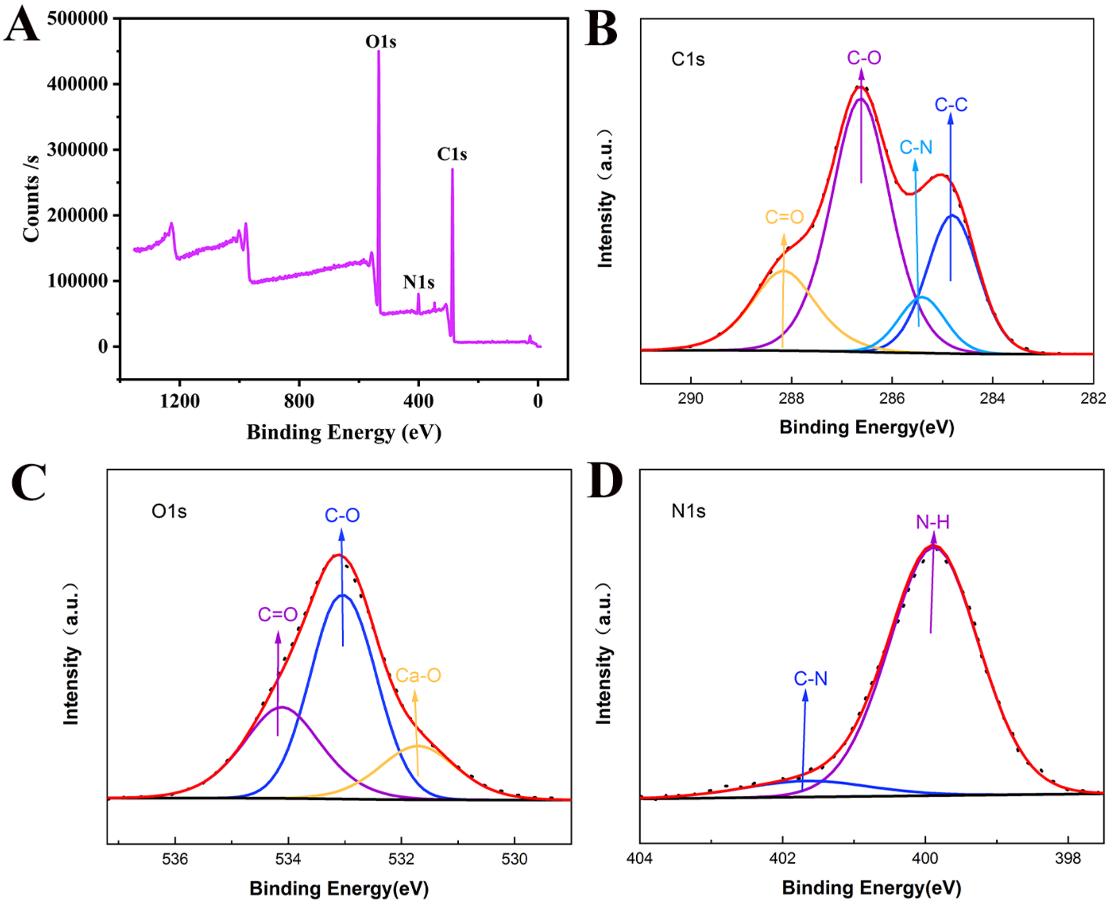

Figure S6. (A) XPS spectrum of CDs. High-resolution XPS peaks of CDs: (B) C1s; (C) O1s; (D) N1s.

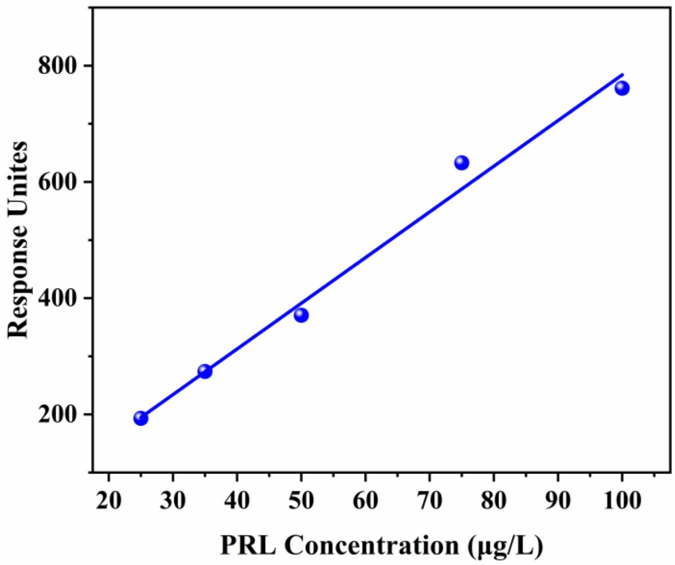

Figure S7. Standard curves of different concentrations of PRL.

Table S1. Dynamic model fitting of the cumulative release curve

Model	Temperature	Expression	Equations	R^2	RSS
**Zero order**	37℃	$Q = K_0t + Q_0$	Q=1.096t+14.583	0.8219	677.50
	60℃		Q=1.127t+33.436	0.6619	1686.94
	80℃		Q=1.1962t+40.090	0.5743	2417.67
**First order**	37℃	$\ln(1-0.01Q) = K_0t + Q_0$	ln(1-0.01Q)=-0.017t-0.151	0.8918	0.096
	60℃		ln(1-0.01Q)=-0.026t-0.412	0.8505	0.302
	80℃		ln(1-0.01Q)=-0.029t-0.542	0.8029	0.551
**Higuchi**	37℃	$Q = K_0 t^{1/2} + Q_0$	$Q=9.261t^{1/2}+11.436$	0.8973	422.87
	60℃		$Q=9.868t^{1/2}+17.775$	0.8800	598.68
	80℃		$Q=9.657t^{1/2}+24.245$	0.7864	1142.44
**Weibull**	37℃	$\ln\ln[100/(100-Q)] = K_0\ln t - Q_0$	lnln[100/(100-Q)=0.754lnt-2.681	0.9935	0.056
	60℃		lnln[100/(100-Q)=0.42lnt-1.179	0.9968	0.008
	80℃		lnln[100/(100-Q)=0.391lnt-0.901	0.9885	0.027
**Hixcon-Crowell**	37℃	$(100 - Q)^{1/3} = - K_0t + Q_0$	$(100 - Q)^{1/3}=-0.023t+4.409$	0.8541	0.205
	60℃		$(100 - Q)^{1/3}=-0.029t+4.403$	0.7692	0.595
	80℃		$(100 - Q)^{1/3}=-0.0323t+3.882$	0.6994	0.989
